# ProBLM Web Server: Protein and Membrane Placement and Orientation Package

**DOI:** 10.1155/2014/838259

**Published:** 2014-07-14

**Authors:** Taylor Kimmett, Nicholas Smith, Shawn Witham, Marharyta Petukh, Subhra Sarkar, Emil Alexov

**Affiliations:** ^1^Department of Computer Science, Clemson University, Clemson, SC 29634, USA; ^2^Computational Biophysics and Bioinformatics, Department of Physics, Clemson University, Clemson, SC 29634, USA

## Abstract

The 3D structures of membrane proteins are typically determined without the presence of a lipid bilayer. For the purpose of studying the role of membranes on the wild type characteristics of the corresponding protein, determining the position and orientation of transmembrane proteins within a membrane environment is highly desirable. Here we report a geometry-based approach to automatically insert a membrane protein with a known 3D structure into pregenerated lipid bilayer membranes with various dimensions and lipid compositions or into a pseudomembrane. The pseudomembrane is built using the Protein Nano-Object Integrator which generates a parallelepiped of user-specified dimensions made up of pseudoatoms. The pseudomembrane allows for modeling the desolvation effects while avoiding plausible errors associated with wrongly assigned protein-lipid contacts. The method is implemented into a web server, the ProBLM server, which is freely available to the biophysical community. The web server allows the user to upload a protein coordinate file and any missing residues or heavy atoms are regenerated. ProBLM then creates a combined protein-membrane complex from the given membrane protein and bilayer lipid membrane or pseudomembrane. The user is given an option to manually refine the model by manipulating the position and orientation of the protein with respect to the membrane.

## 1. Introduction

Membrane proteins are a significant fraction of proteins in the cell [1, 2]. They serve as ion channels [3], ion pumps [4], and transporters [5] among other functions [6]. Due to the importance of membrane proteins, they are one of the primary targets for drug discovery [7, 8]. Although the abovementioned functions are distinctively different [9], they are all performed as the proteins are within membrane environment, indicating that in many cases the membrane is an essential component needed for their proper function.

Revealing the details of the corresponding biological processes occurring in membrane proteins requires knowledge of their 3D structures. However, experimentally determining 3D structures of transmembrane proteins in the presence of the surrounding lipid bilayer environment is very difficult, because they cannot be easily crystallized in 3D crystal lattice [10] and is the reason that relatively fewer membrane protein structures are available as compared to soluble proteins [11]. Typically, membrane proteins are crystallized (sp) in the presence of detergents; however, this approach typically does not provide information about how the protein is situated in the membrane, although it is assumed that the detergent molecules bind to the membrane-buried protein surface. This also means that the alignment and orientation of the membrane protein within the membrane has to be predicted.

There are various approaches for predicting either the transmembrane region of a protein [12–14] or placing a membrane protein into a membrane [15]. Most of the available molecular dynamics (MD) packages offer tools for inserting a protein into lipid bilayer [16–18]. Of particular interest is Charmm Graphical-user interface (Charmm-GUI) which aids users in placing the membrane protein into various lipid membranes [19, 20]. The visual molecular dynamics (VMD) [21] tool offers many options for building lipid bilayer in conjunction with other information [22–25] and can be used to incorporate a protein into the membrane.

In parallel, databases of membrane proteins and their properties were developed such as the Orientations of Proteins in Membranes (OPM) database [26] and TMBETA-GENOME database [27]. These developments were used to create the PPM webserver which can be used for calculating spatial positions in membranes of membrane proteins [28]. Furthermore, once the protein is properly positioned in the membrane, the overlapping lipids and water molecules must be removed. Different approaches exist, as, for example, the GRIFFIN package [29]. As outlined by the authors, the procedure involves two steps: the first one is to carve out lipid and water molecules from a volume equivalent to that of the protein, and the second step is to relax the system with an implicit grid-based protein force field [29].

While many tools for placing membrane proteins into a lipid bilayer exist as outlined above, it is not entirely clear that they will always arrange the lipids interacting with the protein in the same fashion as the native protein-membrane ensemble. In addition, none of the existing servers allows for implicit membrane and very few for manual adjustment (Table 1). It can be argued that an implicit membrane may be preferable in some cases where the errors associated with mispositioning explicit lipids may be quite large. To address such a possibility, we have developed a simple geometrical procedure to predict the transmembrane axis of a protein (note that for some proteins, specifically for single-spanning alpha-helical transmembrane proteins, the axis may be strongly tilted with respect to the membrane normal and manual adjustment will be needed) using the atomic coordinates of the target membrane protein, and then to place it into a pregenerated explicit or user-defined implicit membrane. The protocol has been implemented into a webserver, the Protein and Bilayer Lipid Membrane (ProBLM) web server, which allows the users to visualize the protein and the membrane and to let the users further manipulate protein position and orientation. In addition, the webserver adds missing atoms and residues not provided in the Protein Data Bank (PDB) [30] file.

## 2. Methods

### 2.1. Overall Algorithm for Protein Placement

The geometrical algorithm has two components aimed at predicting the transmembrane axis of a protein that is either mostly helical or mostly beta-stranded. Below we describe these two approaches separately.

#### 2.1.1. Helical Membrane Proteins

**Figure 1:**
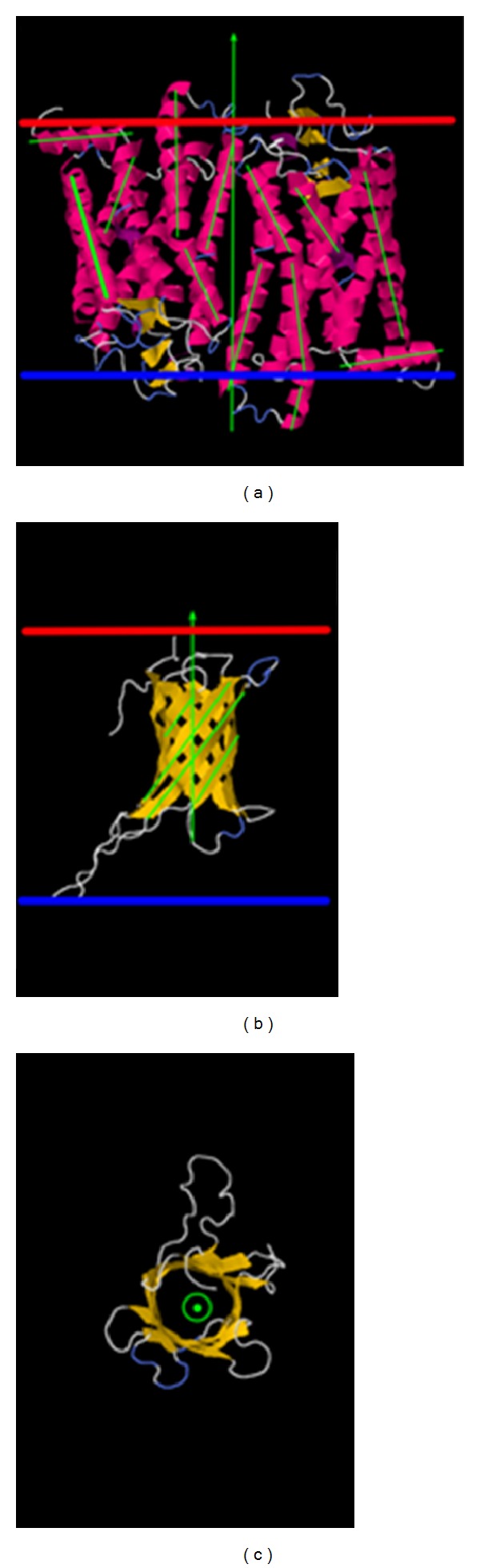
The major axes shown for (a) helical protein (PDB ID: 1GZM) and (b) a beta barrel protein (PDB ID: 2JMM). These major axes are determined by an overall vector ensemble consisting of helical strands or beta sheets. (c) shows a top view of the beta-barrel with the major axis vector pointing through the barrel.

The helical membrane protein usually consists of bundles of helices which may be aligned to each other in a particular direction or it may have a cluster of helices forming the main body of the protein with the addition of another structural segment. Only the main body of the protein is used to find the transmembrane axis. Each helix within the main body is represented as a vector (Figure 1(a)) and then the vectors are summed up in sequential order, but their direction is inverted if the orientation of the vector differs by more than 45 degrees from the previous one. As illustrated in the figure, some helices may be short, do not span the width of the entire membrane, and are followed (in terms of amino acid sequence) by another short helix which goes into the other membrane surfaces. Therefore, the vectors of two such sequential helices will be summed up without the artificial manipulation. However, in the opposite case, a particular helix may run from the top of membrane to the bottom (Figure 1(a)) and the next one (in terms of the amino acid sequence) to be in the opposite direction (from the bottom of the membrane to the top). In this case, the summation is done by first inverting the orientation of the second helix. This approach avoids the “annihilation” of parallel but opposite direction vectors. Once the transmembrane axis (vector) is determined, it is used to reorient the membrane protein such that the vector is parallel to the *z*-axis (by definition, all membranes in the web server, the explicit and implicit, are perpendicular to the *z*-axis). The center of the mass of the main body of the protein is calculated as well and used to position the protein at the origin of the reference frame (0,0, 0). The membrane center by default is also at (0,0, 0). Finally, the short, helical domains are used as an “anchor” point to either the extracellular or cytoplasmic side of the membrane surface to adjust the vertical position of the protein with respect to the membrane. This is done by positioning the short helical domain at the membrane surface.

#### 2.1.2. Beta-Barrel Membrane Proteins

A beta barrel transmembrane protein usually consists of a barrel shape being formed by a coil structure of beta sheets. Vectors are created to connect the openings on each end of the barrel utilizing a geometrical algorithm with specific distance constraints, which creates a major axis vector aligned along the *z*-axis (as shown in Figures 1(b) and 1(c)). Loops are not considered in this process. Similarly as above, the individual vectors associated with each strand are summed up in sequential order, and their orientation is reversed if they are facing opposite membrane surfaces. The final vector is then used to find the transformation matrix to align the entire protein along the *z*-axis. The center of the mass then is used to position the protein at the origin of the reference frame (0,0, 0).

#### 2.1.3. Pseudo-Membrane Generation

The pseudomembrane generation in ProNOI creates collections of uniformly spaced points distributed within a user-defined structure. The object generation currently supports the basic shapes of cylinders, parallelepipeds, cones, and spheres but only the parallelepiped is used in the ProBLM server. By using input vectors and scalars, the program creates uniform, solid objects containing evenly spaced pseudoatoms subject to predefined density parameter. It also redistributes atoms in the volume to ensure that no discontinuities occur at the edges of the object. The output is then printed in PDB format for later use. For use in the ProBLM web server; users define the pseudomembrane by providing three numerical values which represent the width, height, and depth of a parallelepiped (box) which is then translated to the three-vector format required by ProNOI for the parallelepiped. The distance between the atoms of the artificial membrane is predefined as 2.0 Angstroms.

#### 2.1.4. Protein-Membrane Overlap

The protein is then placed with its predicted orientation within the membrane; however, care needs to be taken as atoms from the protein and membrane may overlap. The overlapping atoms of the lipids in the membrane need to be removed which is done during the refinement phase. A lipid is deleted if more than 10% of its atoms are within a cut-off distance of 2 Å. If further analysis and refinement is required, the user can use the entire protein-membrane system in a molecular dynamics relaxation or use other techniques to relax atomic clashes as done in the GRIFFIN package [29].

### 2.2. The Server, Refinement, and Visualization

#### 2.2.1. Web Server Capabilities (Figure 2)

The first element of user input is the protein which is to be embedded in a membrane. The user uploads a PDB file which will automatically be fixed (missing atoms and residues will be added to the protein structure, assuming default protonation states at pH 7). The next step is to define the membrane in which to embed the protein. For this reason, there are three options. First, the user may upload their own membrane PDB file. Second, the user may choose to generate an artificial membrane by specifying the width, height, and depth of a box which will define the custom membrane. The third option is to choose from a list of pregenerated membranes of various sizes and composition (POPC/POPE - Phosphatidylcholine/Phosphatidylethanolamine and various sizes ranging from 50 to 100 Å). Once the protein and membrane choices are made, the user is automatically taken to the next phase, which allows for real-time visualization and refinement in the form of adjustment sliders. Once the user is satisfied with the results, they can download all files to a local machine for further work.

#### 2.2.2. Visualization

The results are presented in real-time visualization software, Jmol, where the user can visually check the results before downloading or using the refinement tools. The visualization can aid the user with refinement by providing immediate, visual feedback of what the refinement phase will do.

#### 2.2.3. Refinement

The refinement phase of the online server package allows the user to adjust the position of the membrane protein in three directions. The three available adjustment directions are positions *z*, *z*-*x* axis angle *θ*, and *x*-*y* axis angle *φ*. These three directions give the user full control over a more suitable orientation that may be specific to their protein's function, along with various other scenarios (i.e., special functioning loops that hang on a specific side of the membrane, important helices that play crucial roles in connecting other peripheral proteins, etc.). Once the user is satisfied with the results, the resulting combined structures are provided instantly in PDB format, which can then be used for further computational analysis.

#### 2.2.4. Nonmembrane Proteins and Failures

The web server can also be used to place nonmembrane protein into a membrane. This may be used to model protein trafficking across the membrane or to compare the water versus membrane environment for the selected cases. In addition, some membrane proteins with complex topology may not be resulting in correct orientation with respect to the membrane. These cases will be automatically detected and the user will receive warning message. The protein still will be positioned within the membrane and situated at the origin (0,0, 0), but the orientation will be achieved manually by the user using the sliders.

## 3. Results and Discussion

**Figure 3:**
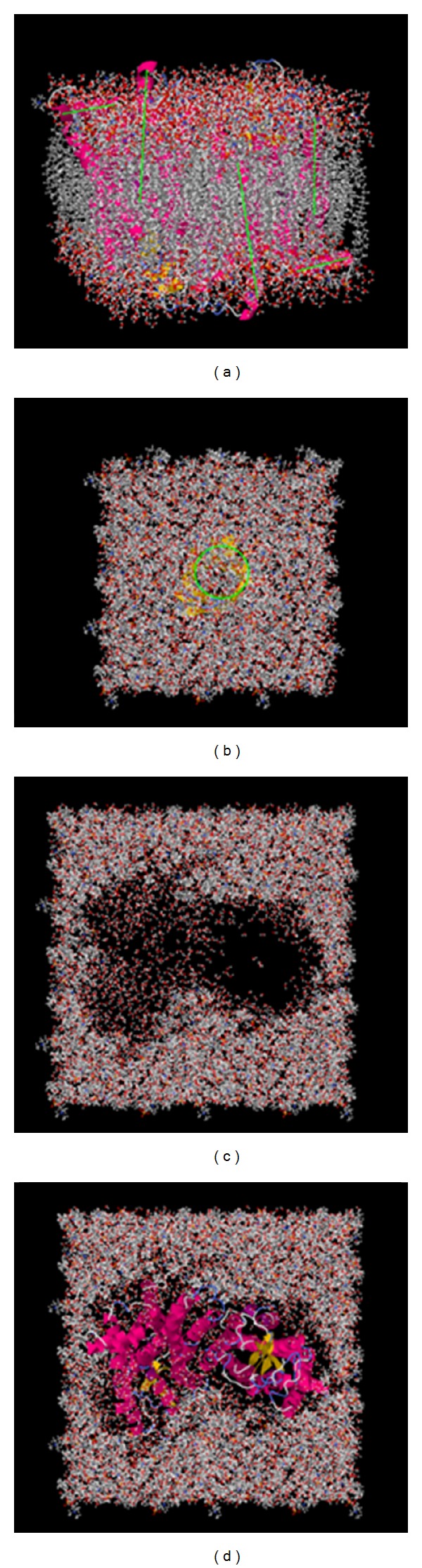
(a) Helical protein (PDB ID: 1GZM), (b) a beta barrel protein (PDB ID: 2JMM) oriented within a 75 × 75 Å membrane. The geometric features of each scenario are highlighted in green. (c) shows the hole where atomic/lipid deletion occurred, and (d) shows the protein placed within the membrane.

Figure 3 shows two typical results from ProBLM (helical and beta-barrel proteins). Before the output is downloaded, the user has the option to refine and visualize the structure. The results are given for immediate download, and the user has the option to download one or both of the structures: (a) combined structure with no deletion of atoms/lipids, and/or (b) the combined structure with atom/lipid deletion.

Depending on the size of the membrane protein being inserted into membrane bilayer, the user can select pregenerated membranes with various dimensions. It is advisable, for small membrane proteins to use membranes with small dimensions and thus to reduce the computational cost for the algorithm that will be using protein-membrane complex output by ProBLM. At the same time, it is advisable as well to have several lipid layers between protein edges and the end of the membrane slab to avoid unwanted edge effects. Thus, for large membrane proteins, one should use membranes with large dimensions. It should be mentioned that the protocol used to relax protein-membrane atomic clashes is geometrically based and does not change the protein or membrane conformation. More sophisticated users may want to download the protein-membrane complex without clashing lipids removed and perform their own refinement procedure(s).

**Figure 4:**
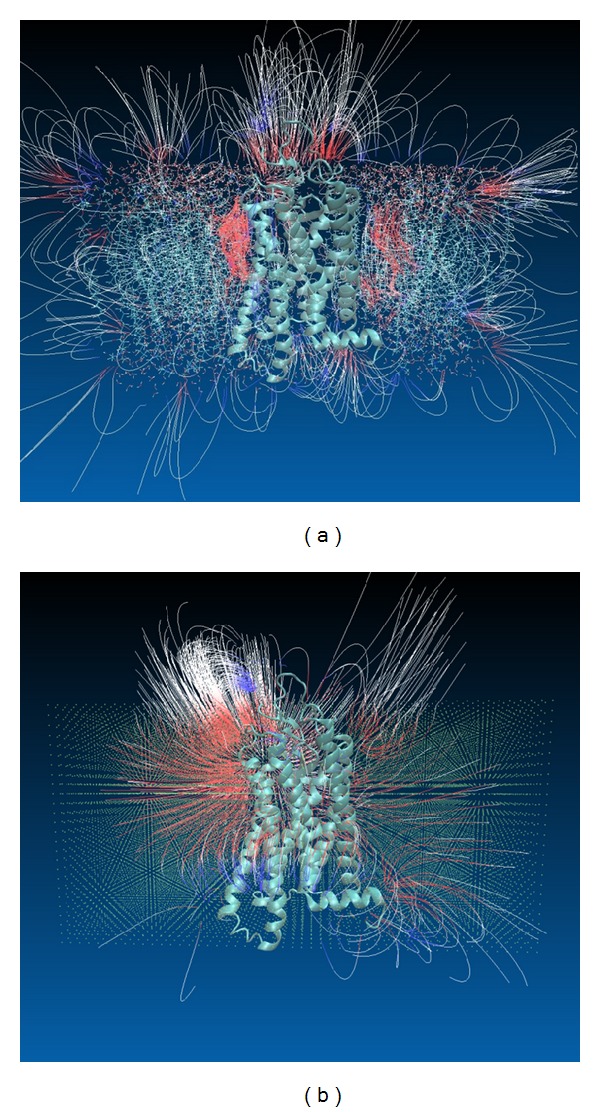
The electrostatic field lines generated with VMD for potential map calculated with DelPhi for explicit and implicit membranes. Color codes are −0.5 and +0.5 for red and blue, respectively. (a) Explicit membrane, (b) implicit membrane.

One of the main applications of protein-membrane complexes generated by ProBLM is expected to be in modeling macromolecular electrostatics. Once the coordinate file is generated, typically it is subjected to modeling procedure that assigns charge and radius to each atom in the complex according to particular force field parameters. Then the electrostatic potential is calculated by solving the Poisson-Boltzmann equation [31]. The result of the calculations is expected to be quite sensitive to the positions of charged atoms and their radii. Due to this, it can be anticipated that the electrostatic potential distributions obtained with explicit and implicit membranes are different. To check such a possibility and to illustrate the electrostatic field distribution in explicit and implicit membranes, a helical protein, PDB ID 1GZM, was inserted by ProBLM in explicit POPC membrane with dimensions 50 × 100 Å. Then the same protein was inserted in implicit membrane with the same dimensions and thickness 50 Å (equivalent to the thickness of explicit membrane). Although each lipid atom in the POPC lipid carries a partial charge, the POPC has zero net charges resulting in zero net charges associated with explicit membrane. To account for this, the implicit membrane pseudoatoms were kept neutral as well (in the case of modeling membrane with nonzero net charges, one can assign appropriate charges to membrane pseudoatoms accordingly and this option has not been explicitly provided in the server and has to be done by the user after obtaining the structure file). Then, each complex was entered into the DelPhi package [32] in order to obtain the distribution of the electrostatic potential. The resulting potential map was then visualized with VMD [21]. The results are shown in Figure 4. One can see that overall electrostatic features are preserved in the implicit membrane, but they are much smoother than in the explicit membrane. The strong negative potential at the top of the protein and on the sides is seen in both cases; however, in explicit membrane it is much more localized. Without knowing the wild-type protein-lipid interactions, one may prefer the implicit membrane results, since the obtained potential and electrostatic filed distributions are smoother and not so much sensitive to ambiguities of the membrane details and protein exact position within membrane.

Additional test was carried out by computing pKa's of ionizable groups of a membrane protein, the bacteriorhodopsin (BR), embedded in explicit and implicit membrane (see previous works with explicit or without membrane [33–37]). The coordinate file was downloaded from Protein Data Base (PDB) [38, 39], PDB ID 1QHJ.pdb [40], and the entire BR trimmer was inputted to ProBLM server. The protein-membrane file with explicit lipids was generated by incorporating the BR trimmer into POPC membrane with dimensions 100 × 100 Å. The combined file, the BR trimmer and implicit membrane, was created using the ProBLM option of generating atomic-style presentation of parallelepiped (box) with the same dimensions being explicit membrane. Pseudo-atoms were kept uncharged to reflect the fact that POPC membrane carries zero net charge. Both structural files were subjected to Multi-Conformation Continuum Electrostatics (MCCE) [41–43] package to calculate the pKa's of ionizable groups. Default parameters were used. The pKa's were averaged over the three molecules within the trimmer (see Table 1S in Supplementary Material available online at http://dx.doi.org/10.1155/2014/838259). The overall standard error (STER) between the pKa's calculated with explicit and implicit membrane is 0.22 pK units, which indicates that the effect of membrane presentation is negligibly small. The most effected residues are the residues situated at the border between the protein and membrane surfaces. Among them, the pKa of Glu 166 is predicted to be 2.0 in the system with explicit membrane and to be 8.1 in case of implicit membrane due to different packing. However, Glu 166 is not known to be functionally important and therefore this discrepancy is not crucial in modeling the reactions in BR. In contrast, the pKa's of all functionally important groups, which are buried in the BR, were calculated to be practically identical for explicit and implicit membrane and to be in very good agreement with experimental data (Table 1S). This finding provides additional support for the claim that implicit membrane model is a good alternative for the explicit membrane.

## 4. Conclusion

The development of a webserver, the ProBLM webserver, is reported, which is aimed at assisting researchers in generating protein-membrane complexes. The ProBLM is unique since it provides the option for the protein to be embedded into explicit and implicit membranes. In addition, it allows the user to manually manipulate the position and orientation of the protein within the membrane. The ProBLM web server is available at http://compbio.clemson.edu/problm_webserver.

## Supplementary Material

The Supplementary Material shows calculated pKa's of ionizable groups of bacteriorhodopsin with explicit and implicit membrane. Available experimental data is provided as well. The calculations were done with Multiple Conformation Continuum Electrostatics (MCCE) package.

## Figures and Tables

**Figure 2:**
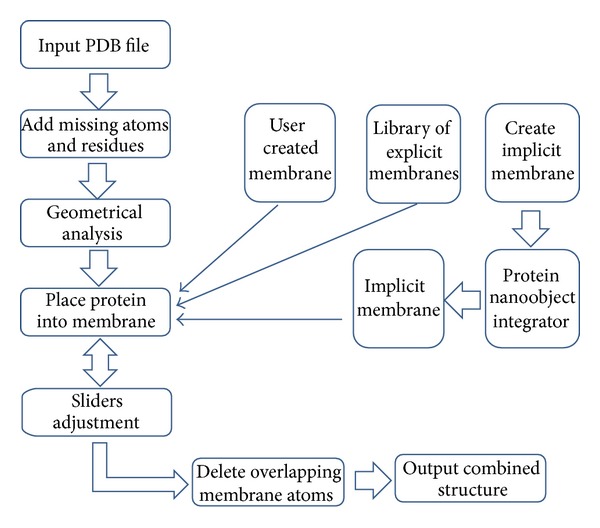
Flowchart of the ProBLM algorithm and web-server implementation.

**Table 1:** Summary of the main differences and similarities between ProBLM and other widely used servers or tools.

Server	Membrane presentation	Manual adjustment	Overlap with lipids treatment
Charmm-GUI	Explicit	Yes	MD relaxation
VMD	Explicit	No	relaxation
PPM	Explicit	No	NA
ProBLM	Explicit and implicit	Yes	Distance cutoff

## References

[1] Pedersen SK, Harry JL, Sebastian L (2003). Unseen proteome: Mining below the tip of the Iceberg to find low abundance and membrane proteins. *Journal of Proteome Research*.

[2] Wallin E, von Heijne G (1998). Genome-wide analysis of integral membrane proteins from eubacterial, archaean, and eukaryotic organisms. *Protein Science*.

[3] Zhong YS, Wang J, Liu WM, Zhu YH (2013). Potassium ion channels in retinal ganglion cells (review). *Molecular Medicine Reports*.

[4] Blostein R (1989). Ion pumps. *Current Opinion in Cell Biology*.

[5] de Koning H, Diallinas G (2000). Nucleobase transporters (review). *Molecular Membrane Biology*.

[6] Besschetnova TY, Montefusco DJ, Asinas AE, Shrout AL, Antommattei FM, Weis RM (2008). Receptor density balances signal stimulation and attenuation in membrane-assembled complexes of bacterial chemotaxis signaling proteins. *Proceedings of the National Academy of Sciences of the United States of America*.

[7] Doshi D, Marx SO (2009). Ion channels, transporters, and pumps as targets for heart failure therapy. *Journal of Cardiovascular Pharmacology*.

[8] Sissung TM, Gardner ER, Gao R, Figg WD (2008). Pharmacogenetics of membrane transporters: a review of current approaches. *Methods in Molecular Biology*.

[9] Gadsby DC (2009). Ion channels versus ion pumps: the principal difference, in principle. *Nature Reviews Molecular Cell Biology*.

[10] Lacapère J, Pebay-Peyroula E, Neumann J, Etchebest C (2007). Determining membrane protein structures: still a challenge!. *Trends in Biochemical Sciences*.

[11] Lundstrom K (2004). Structural genomics on membrane proteins: mini review. *Combinatorial Chemistry and High Throughput Screening*.

[12] Yu DJ, Shen HB, Yang JY (2012). SOMPNN: an efficient non-parametric model for predicting transmembrane helices. *Amino Acids*.

[13] Shen H, Chou JJ (2008). Membrain: improving the accuracy of predicting transmembrane helices. *PLoS ONE*.

[14] Gromiha MM (1999). A simple method for predicting transmembrane *α* helices with better accuracy. *Protein Engineering*.

[15] Tatulian SA, Qin S, Pande AH, He X (2005). Positioning membrane proteins by novel protein engineering and biophysical approaches. *Journal of Molecular Biology*.

[16] Brooks BR, Brooks CL, Mackerell AD (2009). CHARMM: the biomolecular simulation program. *Journal of Computational Chemistry*.

[17] Case DA, Cheatham TE, Darden T (2005). The Amber biomolecular simulation programs. *Journal of Computational Chemistry*.

[18] Christen M, Hünenberger PH, Bakowies D (2005). The GROMOS software for biomolecular simulation: GROMOS05. *Journal of Computational Chemistry*.

[19] Jo S, Lim JB, Klauda JB, Im W (2009). CHARMM-GUI membrane builder for mixed bilayers and its application to yeast membranes. *Biophysical Journal*.

[20] Jo S, Kim T, Iyer VG, Im W (2008). CHARMM-GUI: A web-based graphical user interface for CHARMM. *Journal of Computational Chemistry*.

[21] Humphrey W, Dalke A, Schulten K (1996). VMD: visual molecular dynamics. *Journal of Molecular Graphics*.

[22] Lomize AL, Pogozheva ID, Mosberg HI (2011). Anisotropic solvent model of the lipid bilayer. 2. Energetics of insertion of small molecules, peptides, and proteins in membranes. *Journal of Chemical Information and Modeling*.

[23] Lomize AL, Pogozheva ID, Mosberg HI (2011). Anisotropic solvent model of the lipid bilayer. 1. Parameterization of long-range electrostatics and first solvation shell effects. *Journal of Chemical Information and Modeling*.

[24] Lomize AL, Pogozheva ID, Lomize MA, Mosberg HI (2007). The role of hydrophobic interactions in positioning of peripheral proteins in membranes. *BMC Structural Biology*.

[25] Lomize AL, Pogozheva ID, Lomize MA, Mosberg HI (2006). Positioning of proteins in membranes: a computational approach. *Protein Science*.

[26] Lomize MA, Lomize AL, Pogozheva ID, Mosberg HI (2006). OPM: orientations of proteins in membranes database. *Bioinformatics*.

[27] Gromiha MM, Yabuki Y, Kundu S, Suharnan S, Suwa M (2007). TMBETA-GENOME: database for annotated *β*-barrel membrane proteins in genomic sequences. *Nucleic Acids Research*.

[28] Lomize MA, Pogozheva ID, Joo H, Mosberg HI, Lomize AL (2012). OPM database and PPM web server: resources for positioning of proteins in membranes. *Nucleic Acids Research*.

[29] Staritzbichler R, Anselmi C, Forrest LR, Faraldo-Gómez JD (2011). GRIFFIN: a versatile methodology for optimization of protein-lipid interfaces for membrane protein simulations. *Journal of Chemical Theory and Computation*.

[30] Rose PW, Bi C, Bluhm WF (2013). The RCSB Protein Data Bank: new resources for research and education. *Nucleic Acids Research*.

[31] Baker NA (2004). Poisson-Boltzmann methods for biomolecular electrostatics. *Methods in Enzymology*.

[32] Li L, Li CS, Sarkar J (2012). DelPhi: a comprehensive suite for DelPhi software and associated resources. *BMC Biophysics*.

[33] Onufriev A, Smondyrev A, Bashford D (2003). Proton affinity changes driving unidirectional proton transport in the bacteriorhodopsin photocycle. *Journal of Molecular Biology*.

[34] Spassov VZ, Luecke H, Gerwert K, Bashford D (2001). pKa calculations suggest storage of an excess proton in a hydrogen-bonded water network in bacteriorhodopsin. *Journal of Molecular Biology*.

[35] Sampogna RV, Honig B (1996). Electrostatic coupling between retinal isomerization and the ionization state of Glu-204: a general mechanism for proton release in bacteriorhodopsin. *Biophysical Journal*.

[36] Sampogna RV, Honig B (1994). Environmental effects on the protonation states of active site residues in bacteriorhodopsin. *Biophysical Journal*.

[37] Song Y, Mao J, Gunner MR (2003). Calculation of proton transfers in bacteriorhodopsin bR and M intermediates. *Biochemistry*.

[38] Berman H, Henrick K, Nakamura H, Markley JL (2007). The worldwide Protein Data Bank (wwPDB): ensuring a single, uniform archive of PDB data. *Nucleic Acids Res*.

[39] Kouranov A, Xie L, de la Cruz J (2006). The RCSB PDB information portal for structural genomics. *Nucleic Acids Research*.

[40] Belrhali H, Nollert P, Royant A (1999). Protein, lipid and water organization in bacteriorhodopsin crystals: a molecular view of the purple membrane at 1.9 Å resolution. *Structure*.

[41] Zhang J, Gunner MR (2010). Multiconformation continuum electrostatics analysis of the effects of a buried asp introduced near heme a in rhodobacter sphaeroides cytochrome c Oxidase. *Biochemistry*.

[42] Song Y, Gunner MR (2009). Using multiconformation continuum electrostatics to compare chloride binding motifs in alpha-amylase, human serum albumin, and Omp32. *Journal of Molecular Biology*.

[43] Georgescu RE, Alexov EG, Gunner MR (2002). Combining conformational flexibility and continuum electrostatics for calculating pK_a_s in proteins. *Biophysical Journal*.

