# Supplementary material for: ProBLM Web Server: Protein and Membrane Placement and Orientation Package
Source: Comput Math Methods Med. 2014 Jul 14;2014:838259. doi: 10.1155/2014/838259 (PMC4122144; doi:10.1155/2014/838259)
Supplement: Supplementary file 1 — The Supplementary Material shows calculated pKa's of ionizable groups of bacteriorhodopsin with explicit and implicit membrane. Available experimental data is provided as well. The calculations were done with Multiple Conformation Continuum Electrostatics (MCCE) package. [file 838259.f1.pdf]

Supplementary material:

Taylor Kimmett, Nicholas Smith, Shawn Witham, Marharyta Petukh, Subhra Sarkar, Emil Alexov

Table 1S. Calculated pKa's of ionizable groups of bacteriorhodopsin with explicit and implicit membrane. pKa predicted to be larger than 14.0 was truncated to 14, while pKa predicted to be smaller than 0.0 was marked as 0.

Residue	#	pKa_experim	Explicit membrane	Implicit membrane
ARG	7		13.648	13.29
GLU	9		10.337	10.104
TYR	26		9.586	10.379
LYS	30		10.128	9.901
ASP	36		1.78	3.028
ASP	38		3.185	2.001
LYS	40		10.135	14
LYS	41		12.583	13.566
TYR	43		14	14
TYR	57		14	14
TYR	64		13.071	12.968
GLU	74		4.33	4.452
TYR	79		14	14
ARG	82	>13.8	14	14
TYR	83		14	14
ASP	85	2.6	1.593	0
ASP	96	>12.0	14	14
ASP	102		5.105	3.571
ASP	104		0.836	1.722
ASP	115	> 9.5	9.064	8.92
LYS	129		10.93	10.605
TYR	131		8.278	10.73
TYR	133		10.726	10.346
ARG	134		14	14
TYR	147		12.459	12.847
TYR	150		10.366	10.251
LYS	159		12.539	12.36
GLU	161		4.078	3.779
ARG	164		12.823	14

**GLU**	**166**		**1.98**	**8.101**
**LYS**	**172**		**10.553**	**8.964**
**ARG**	**175**		**11.594**	**12.436**
**TYR**	**185**		**14**	**14**
**GLU**	**194**		**0**	**0**
**GLU**	**204**		**10.906**	**13.01**
**ASP**	**212**	**< 2.5**	**0**	**0**
**LYS**	**216**	**>12.0**	**14**	**14**
**ARG**	**225**		**14**	**13.339**
**ARG**	**227**		**14**	**14**
**GLU**	**232**		**3.414**	**4.576**
